# Functional Characterization of Cooperating MGA Mutations in RUNX1::RUNX1T1 Acute Myeloid Leukemia

**DOI:** 10.21203/rs.3.rs-3315059/v1

**Published:** 2023-09-22

**Authors:** Jeffery Klco, Melvin Thomas, Wenqing Qi, Michael Walsh, Jing Ma, Tamara Westover, Sherif Abdelhamed, Lauren Ezzell, Chandra Rolle, Emily Xiong, Wojciech Rosikiewicz, Beisi Xu, Shondra Pruett-Miller, Allister Loughran, Laura Janke

**Affiliations:** St. Jude Children’s Research Hospital; St. Jude Children’s Research Hospital; St. Jude Children’s Research Hospital; St. Jude Childrens Hospital; St. Jude Children’s Research Hospital; St. Jude Children’s Research Hospital; Seagen; St. Jude Children’s Research Hospital; St. Jude Children’s Research Hospital; St. Jude Children’s Research Hospital; St. Jude Children’s Research Hospital; St Jude Children’s Research Hospital; St. Jude Children’s Research Hospital; St. Jude Children’s Research Hospital; St. Jude Children’s Research Hospital

**Keywords:** MGA, RUNX1:RUNX1T1, AML, MYC regulation, ncPRC1.6

## Abstract

MGA (Max-gene associated) is a dual-specificity transcription factor that negatively regulates MYC-target genes to inhibit proliferation and promote differentiation. Loss-of-function mutations in *MGA* have been commonly identified in several hematological neoplasms, including acute myeloid leukemia (AML) with *RUNX1::RUNX1T1,* however, very little is known about the impact of these *MGA* alterations on normal hematopoiesis or disease progression. We show that representative *MGA* mutations identified in patient samples abolish protein-protein interactions and transcriptional activity. Using a series of human and mouse model systems, including a newly developed conditional knock-out mouse strain, we demonstrate that loss of *MGA* results in upregulation of MYC and E2F targets, cell cycle genes, mTOR signaling, and oxidative phosphorylation in normal hematopoietic cells, leading to enhanced proliferation. The loss of *MGA* induces an open chromatin state at promotors of genes involved in cell cycle and proliferation. *RUNX1::RUNX1T1* expression in Mga-deficient murine hematopoietic cells leads to a more aggressive AML with a significantly shortened latency. These data show that MGA regulates multiple pro-proliferative pathways in hematopoietic cells and cooperates with the RUNX1::RUNX1 T1 fusion oncoprotein to enhance leukemogenesis.

## Introduction

Max-gene associated (MGA) is a transcription factor that uses an n-terminal T-box domain and a c-terminal MYC-like basic helix-loop-helix (bHLH) domain to regulate MAX-network and T-box family targets^1^. MGA interacts with MAX, L3MBTL2, E2F6, and PCGF6 as a part of the non-canonical polycomb repressive complex (ncPRCI .6)^2^ and is required for PRC1.6 complex formation. In non-hematopoietic cells, the recruitment of MGA to T-box and MAX-network target genes was shown to result in the deposition of repressive histone marks such as H2AK119ub1 and H3K27me3^2,3^. MGA is required for embryogenesis and its depletion leads to aberrant embryonic stem cell differentiation and embryonic lethality^4–6^.

Heterozygous somatic alterations of *MGA,* the most common of which lead to loss-of-function truncation/deletion of the MYC-like bHLH domain, occur in 5% of all cancers and are commonly seen in lung adenocarcinoma, endometrial carcinoma, and colorectal cancer^7–10^. The loss of *MGA* in lung adenocarcinoma cells, which occurs in ~ 8% of lung adenocarcinoma patients, was shown to lead to an upregulation of MYC- and E2F6-arget genes, resulting in an increase in cancer cell proliferation and invasiveness^11,12^. *MGA* has also been identified as a common genetic alteration in hematological neoplasms, including acute myeloid leukemia (AML), chronic lymphocytic leukemia (CLL), natural killer/T-cell lymphoma, B-cell acute lymphoblastic leukemia (B-ALL), and T-cell acute lymphoblastic leukemia (T-ALL)^13–18^. In particular, we previously identified *MGA* mutations as recurrent alterations in AMLs with *RUNX1::RUNX1T1* fusions^17^. Likewise, similar mutations have been observed in AMLs with *KMT2A-PTD*^16^.

Despite the recurrence of these mutations, the molecular role of MGA in normal hematopoiesis, as well as in hematopoietic malignancies, has been understudied. This is partly due to the embryonic lethality of in vivo *MGA* deficiency models, which we circumvented in this study by developing a conditional knockout mouse model. Harnessing a multidisciplinary approach to characterize the hematopoietic function of MGA in both human and mouse cells, we establish that MGA loss leads to an increase in proliferation via the upregulation of several cell cycle pathway genes and results in a shortened latency in a mouse model of *RUNX1::RUNX1T1*-driven leukemia.

## Results

**Figure 1:**
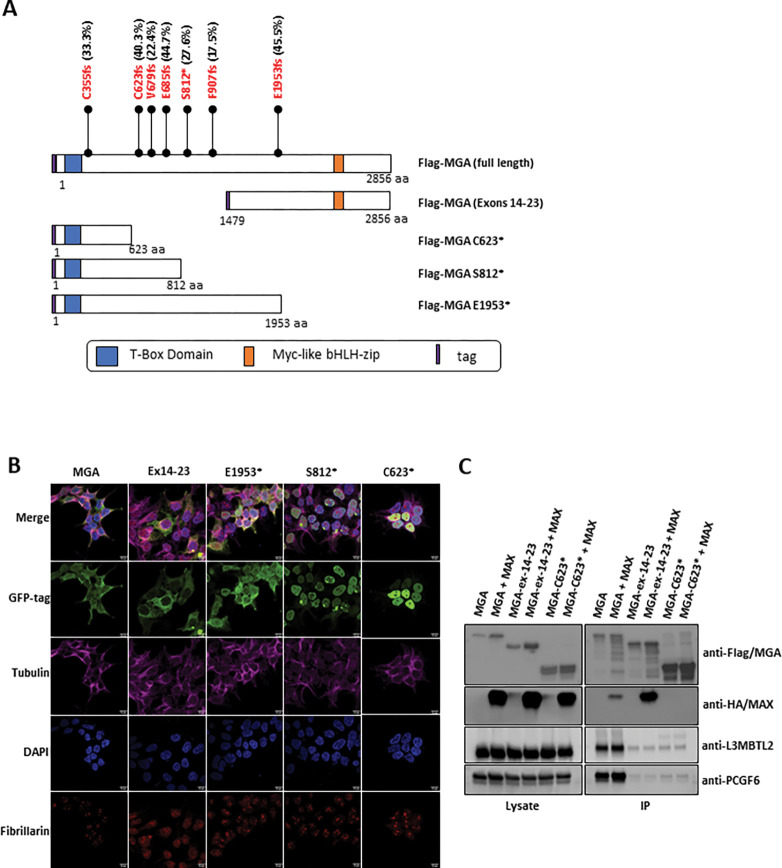
MGA mutations lead to loss of molecular function. **A**. Schematic of full-length MGA with protein domains and mutations identified in *RUNX1::RUNX1T1* AMLs and an experimentally-designed truncation of the T box domain^17^. Tag = GFP or Flag. **B**. Variant allele frequencies of MGA mutations identified in *RUNX1::RUNX1T1* patients called by whole exome sequencing and RNA read counts, adapted from (Faber et al, 2016)^17^. **C**. Immunofluorescent confocal microscopy of HEK293T cells expressing GFP-agged MGA and the indicated truncations. MGA is labeled with eGFP (green), fibrillarin with Alex Fluor568 (red), tubulin with Alex Fluor647 (cyan), and nucleus with DAPI (blue). Images were collected on a Nikon C2 laser scanning confocal microscope using a 60X oil immersion optical lens. **D**. Western blot analysis of immunoprecipitation of Flag-tagged MGA, HA-tagged-MAX, and indicated truncations in HEK293T cells probed for components of the ncPRC1.6 complex.

The reported mutations of *MGA* in pediatric *RUNX1::RUNX1T1* leukemias are heterozygous, somatic mutations at variable variant allele frequencies and result in the deletion of the MYC-like bHLH domain, potentially resulting in a protein with altered or abolished function (Fig. 1A)^17^. We first assessed the protein localization of MGA, including full-length MGA (WT-MGA) and 3 MGA truncations (MGA p.E1953*, MGA p.S812*, and MGA p.C623*) identified in patient samples, by transfecting GFP fusion expression vectors in HEK293T cells (Fig. 1A). We also included an n-terminus truncation which deletes the T-box domain; this construct expresses exons 14–23, including the bHLH domain, to serve as control along with WT-MGA. WT-MGA was localized in both the cytoplasm and nucleus, while the MGA-ex14–23 control was exclusively found in the cytoplasm, which suggests the n-terminal region is required for nuclear localization (Fig. 1B). The MGA p.S812* and MGA p.C623* mutant proteins were expressed exclusively in the nucleus, while the MGA p.E1953* truncation maintained normal localization in both the nucleus and cytoplasm. The retained nuclear localization of MGA truncations suggests these mutations could still be functional.

We then performed co-immunoprecipitation (Co-IP) assays followed by western blot to determine if MGA truncations interacts with components of the ncPRC1.6 complex. We co-expressed flag-tagged WT-MGA, MGA-ex14–23, and MGA p.C623* with HA-tagged MAX in HEK293T cells and performed IP-western blots. We found that MGA p.C623* does not significantly bind L3MBTL2, PCGF6, or MAX, suggesting it loses its interaction with the ncPRC1.6 complex (Fig. 1C). Although nuclear MGA p.C623* does not interact with components of the ncPRC1.6 complex, it still could interact with the genome and hamper the genomic binding of MGA and the ncPRC1.6 complex in a dominant-negative manner at MGA binding sites. To examine this, we expressed HA-tagged MGA p.C623* in MOLM-13 cells and observed a global loss in genomic binding by Cleavage Under Targets and Release using Nuclease (CUT&RUN) assays. In addition to this genome-wide decrease in binding of the MGA mutant, binding was abolished at defined MGA target sites such as *CCND2*(**Supp.** Figure **1A&B**).

**Figure 2:**
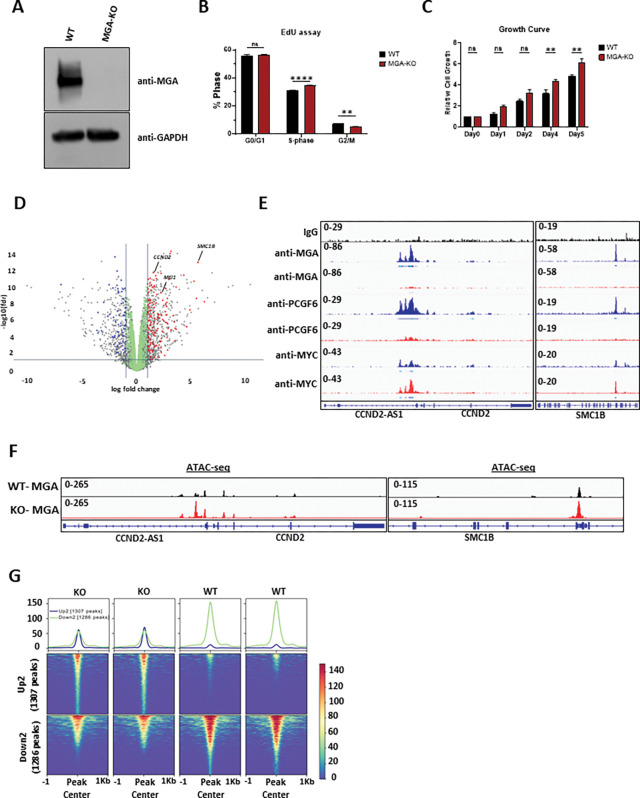
Loss of MGA *in vitro* promotes cell growth. **A**. Western blot analysis of MGA in WT and MGA-KO cells. **B**. Flow cytometric analysis of WT (n=3) and MGA-KO (n=3) MOLM-13 cells treated with EdU for 2hrs and stained with DAPI. **C**. Cell growth assay of WT and MGA-KO MOLM-13 cells over time. Statistics: Two-way ANOVA with Sidak’s multiple comparisons test; error bars indicate the standard error of the mean from three or more biological replicates. **D**. Volcano plot of differentially expressed genes (DEGs) from MGA-KO MOLM-13 cells. Red & Blue points represent upregulated and downregulated genes, respectively, and that have wild-type MGA binding at their promoter-TSS as determined by CUT&RUN. Green points represent nonsignificant transcriptional changes with MGA binding at promoter-TSS. **E**. CUT&RUN coverage plots of MGA, PCGF6, and MYC occupancy on *CCND2* and *SMC1B* in WT (blue) and MGA-KO (red) MOLM-13 cells. **F**. Chromatin occupancy at CCND2 and *SMC1*Bpromoters from ATAC-Seq in WT (black) and MGA-KO (red) MOLM-13 cells. **G**. Replicate tornado plots of ATAC-Seq showing the signal enrichment at the most stringent threshold - Up2 and Down2 (FC > 2, FDR < 0.05). **H**. Venn diagram of differential peaks at open or closed promoters (FDR < 0.05) for differentially expressed genes (q-value < 0.05) in RNA-seq of MGA-KO MOLM-13 cells. Statistics: Venn diagram p-values calculated using hypergeometric distribution.

The loss of interaction with the ncPCR1.6 complex and the loss of DNA binding of MGA p.C623* supports the hypothesis that the truncating mutations of *MGA* identified in patients are loss-of-function mutations, leading to haploinsufficiency. To better understand the impact of MGA loss in hematopoietic cells, we designed a knock-out (KO) model of *MGA* using a CRISPR-based approach in MOLM-13 cells (Fig. 2A). The loss of MGA in these cells led to a slight increase in proliferation (Fig. 2B&C). RNA-sequencing (RNA-seq) analysis showed that the loss of *MGA* led to the upregulation of several genes that play a critical role in proliferation, genome stability, and tumorigenicity, including Cyclin D1 (*CCND1*), Cyclin D2 *(CCND2),* and Structural Maintenance of Chromosomes 1B *(SMC1B)* (Fig. 2D **and Supp**. Figure 1C)^19–21^. Supporting our RNA-seq data, we found that MGA binds to the *CCND2* and *SMC1B* promoters and that MGA deletion reduces PCGF6 binding at these promoters and at the genome-wide level (Fig. 2E **and Supp**. Figure 1D). In contrast, MYC binding is minimally disrupted and potentially enhanced at *CCND2* and *SMC1B* promoters (Fig. 2E, **Supp**. Figure 1D). We also reveal that the loss of MGA resulted in little to no change in the total number of TSS (transcription start site) occupied by H2AK119Ub1, H3K27me3, and H3K4me3. However, its deletion led to an increase in the total number of TSS with the active histone mark of H3K27Ac (**Supp**. Figure 1E**&F**). In addition, the intensity (log2Fold change) of active marks H3K4me3 and H3K27Ac at TSS are significantly higher, while the intensity of repressive mark H3K27me3 is lower in MGA-KO MOLM-13 cells, suggesting that MGA depletion leads to a more active epigenetic landscape (**Supp.** Figure 1G). Supporting these data, ATAC-seq analysis demonstrates that the loss of *MGA* leads to a more open chromatin state at the promoters of *CCND2* and *SMC1B* with minimal global chromatin accessibility changes (Fig. 2F&G). These observations were validated in primary cord blood CD34 +cells immortalized by *RUNX1::RUNX1T1,* in which disruption of *MGA* by CRISPR leads to a reduction of PCGF6 binding to the *CCND2* promoter associated with an overall increase in *CCND2* expression, as well as an increase in self-renewal capacity (**Supp**. Figure 1H-J).

**Figure 3:**
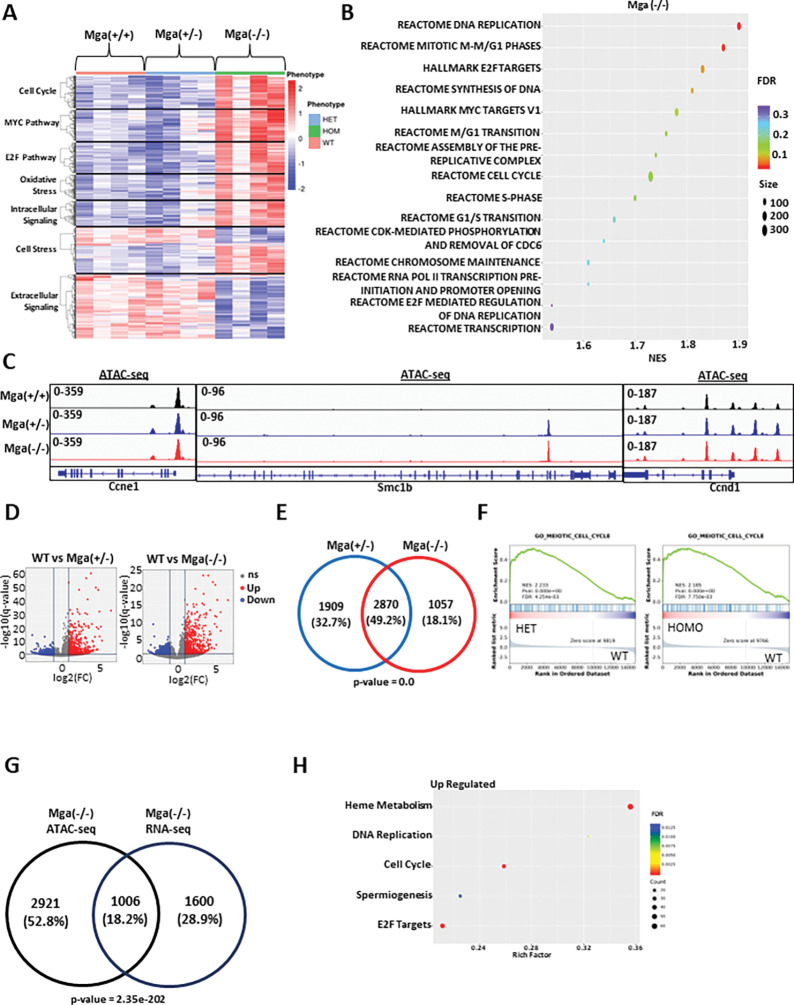
Loss of *Mga* in vivo leads to aberrant cell cycle pathway signaling. **A.** Expression heatmap showing the enrichment of differentially regulated pathways in WT, Mga(+/−), and Mga(−/−) HSPCs isolated from BM. The z-scale is set to between 2 and −2; red=upregulation, blue = downregulation. **B**. Rich factor plots of GSEA indicating upregulated pathways in Mga(−/−) HSPCs. The size of each dot represents gene count and the color represents FDR. **C**. Coverage plots of ATAC-seq analysis of chromatin status at on *Ccne1, Smc1b,* and *Ccnd1* promoters in WT (black), Mga(+/−) (blue), and Mga(−/−) (red) HSPCs. **D**. ATAC-seq volcano plot for Mga(+/−) vs. WT and Mga(−/−) vs. WT. Statistical analysis for ATAC-seq are: Up/Down = log2(FC) > 1 and q-value < 0.05, ns = not significant. **E**. Venn diagram of differentially expressed peaks at open promoters (p-value < 0.05) comparing Mga(+/−) and Mga(−/−) **F**. GSEA plot for Mga(+/−) vs. WT and Mga(−/−) vs. WT. **G**. Venn diagram of differentially expressed genes with increased accessibility (p-value < 0.05) for Mga(−/−) and upregulated genes (p-value < 0.05) in RNA-seq of Mga(−/−) HSPCs. **H** . Rich factor plots showing the differentially regulated pathways of common up/open genes identified in (G) from RNA-seq/ATAC-seq analysis. The size of each dot represents gene count and the color represents FDR. Statistics: Venn diagram p-values calculated using hypergeometric distribution.

Considering that *MGA* loss leads to a cell growth advantage in leukemic cell lines, we wanted to assess the role of MGA in hematopoietic cell development. To this aim, we designed a conditional knock-out model in the C57BL/6 background, in which LoxP sites were introduced that flank exon 3 of *Mga* (**Supp**. Figure 2A). When crossed with Vav1-Cre, this leads to the deletion of exon 3 and the effective deletion of *Mga* in hematopoietic cells. This conditional hematopoietic cell KO model circumvented the known embryonic lethality of homozygous *Mga* loss^6^, as offspring with both heterozygous (Vav1-cre^tg/^+ x Mga^fl/+^ = Mga(+/−)) and homozygous (Vav1-cre^tg/^+ x Mga^fl/fl^ = Mga(−/−)) deletions of *Mga* induced by Vav1-Cre were viable with normal Mendelian ratios. Although the mutations identified in patients are heterozygous, we also established a cohort of mice with homozygous deletion of *Mga* to better define the functional role of *Mga* in hematopoiesis. RNA-seq on lineage- (lin−),cKit + hematopoietic stem and progenitor cells (HSPCs) isolated from the bone marrow (BM) of Mga(+/+) (control), Mga(+/−), and Mga(−/−) mice demonstrated dysregulation of several critical pathways in Mga(−/−) but not in control or Mga(+/−) HSPCs, including the upregulation of Myc and E2F targets and the downregulation of extracellular signaling pathways including JAK-STAT, TGF0, and TNFa signaling (Fig. 3A&B, **Supp**. Figure 2C). Similar to our findings in the MOLM-13 *MGA* knock-out model, Mga(−/−) HSPCs had significant upregulation of cell cycle and proliferation genes, including *Ccne1, Condi, Ccna2, Hdac2,* and *Smc1b* (**Supp**. Figure 2D). To assess the epigenetic changes resulting from the loss of *Mga,* we performed CUT&RUN and ATAC-seq on cKit + HSPCs from these mice. Unfortunately, an anti-murine MGA antibody compatible with CUT&RUN is not available. However, both heterozygous and homozygous deletions of *Mga* led to increases in H3K4me3 and H3K27Ac marks and a decrease in H3K27me3 at the promoters of genes such as *Cone1, Smc1b,* and *Cdk1* (**Supp**. Figure 3A-C). ATAC-seq analysis showed no significant changes in promoter accessibility of *Ccne1* and *Cdk1,* however, the promoter of *Smc1b,* which is closed in control HSPCs, showed significantly more ATAC-seq signals in both Mga(+/−) and Mga(−/−) HSPCs (Fig. 3C). Globally, both heterozygous and homozygous deletions of *Mga* led to a significant increase in open chromatin at gene promoters compared to WT controls (5043 promoters (p < 0.05) and 3984 promoters (p < 0.05), respectively) (Fig. 3D, **Supp**. Figure 3D). There is a ~ 50% overlap of open promoters between Mga(+/−) and Mga(−/−) HSPCs leading to a nearly identical gene set enrichment with both conditions showing a strong enrichment of peaks of promoter regions of cell cycle-related genes (Fig. 3E&F). When comparing the upregulated genes from RNA-seq in Mga(−/−) HSPCs with the enriched ATAC-seq open promoter genes for Mga(−/−), we found an overlap of 1006(18.2%) genes (Fig. 3G). This overlapping gene list is enriched for cell cycle, pro-proliferation, and chromosome stability pathway activation, suggesting that these cells have improved fitness and proliferate faster than controls (Fig. 3H).

**Figure 4:**
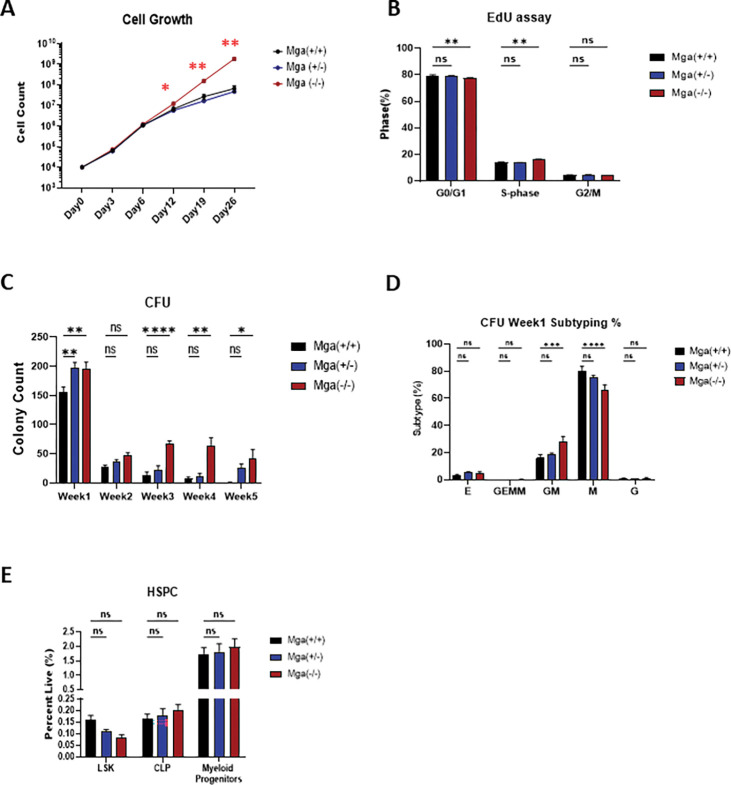
Loss of Mga *in vivo* primes myeloid progenitors for a growth advantage. **A**. Cell growth assay of lineage-negative HSPCs from WT (n=3), Mga(+/−) (n=3), and Mga(−/−) (n=3) mice. **B**. Flow cytometric analysis of lineage-negative HSPCs from WT (n=7), Mga(+/−) (n=7), and Mga(−/−) (n=7) mice treated *ex vivo* with EdU for 2hrs and stained with DAPI. **C**. Colony Forming Units (CFU) showing the total number of colonies from lineage-negative HSPCs from WT (n=9), Mga(+/−) (n=12), and Mga(−/−) (n=9) mice. **D**. Relative distribution of colony subtypes in (**C**), E = erythroid; GEMM = granulocyte, erythrocyte, monocyte, megakaryocyte; GM = granulocyte, monocyte; M = monocyte; G = granulocyte. **E**. Flow cytometric analysis of HSPCs from whole bone marrow harvested from WT (n=6), Mga(+/−) (n=6), and Mga(−/−) (n=7) mice; LSK = lin−, Sca-1+, cKit+; CLP = lin−, Sca-1 lo, cKit-, CD127+; Myeloid Progenitors = lin−, Sca-1-, cKit+. Statistics: (**A**) One-way ANOVA with Bonferroni correction (* p<0.05, ** p<0.01). Error bars indicate the standard error of the mean from three or more biological replicates. (B-E) Two-way ANOVA with Dunnett’s multiple comparisons test.

In addition to these molecular perturbations, loss of Mga in hematopoietic cells also has functional consequences. Mga(−/−) HSPCs have a significant increase in cell growth and proliferation and in self-renewal capacity when compared to controls, while Mga(+/−) HSPCs have only a slight advantage (Fig. 4A–D). These *ex vivo* analyses of *Mga* deficient cells propose that the loss of *Mga* may alter hematopoietic homeostasis. However, Mga(+/−) and Mga(−/−) mice at 3–6 months displayed normal CBCs and demonstrated no defect in the development of B cells (B220+), T cells (CD3e+), or myeloid cells (CD11b+) (**Supp**. Figure 3E-F). Ki67 staining of the B cell, T cell, or myeloid cell populations showed a slight increase in cycling B and T cells but not myeloid cells (**Supp**. Figure 3G). HSPC immunophenotyping by flow cytometry showed no change in the lymphoid progenitor (lin− cKit−, Sca-1^low^), myeloid progenitor (lin−, cKit+) or LSK (lin−, cKit+, Sca-1+) populations from both Mga(+/−) and Mga(−/−) mice (Fig. 4E). Aged control, Mga(+/−), and Mga(−/−) mice (16 months) did not spontaneously develop a hematopoietic malignancy (data not shown). Despite the observed self-renewal phenotype, serial noncompetitive BM transplants of HSPCs isolated from control, Mga(+/−), and Mga(−/−) mice (**Supp**. Figure 4A) lead to no significant changes in WBC counts or in the ability to reestablish mature blood cell production over a 16 week period (**Supp**. Figure 4B-G). However, primary recipient mice that received Mga(−/−) HSPCs had a slight decrease in LSKs and an increase in myeloid progenitor cells (**Supp**. Figure 4B, D&E).

**Figure 5:**
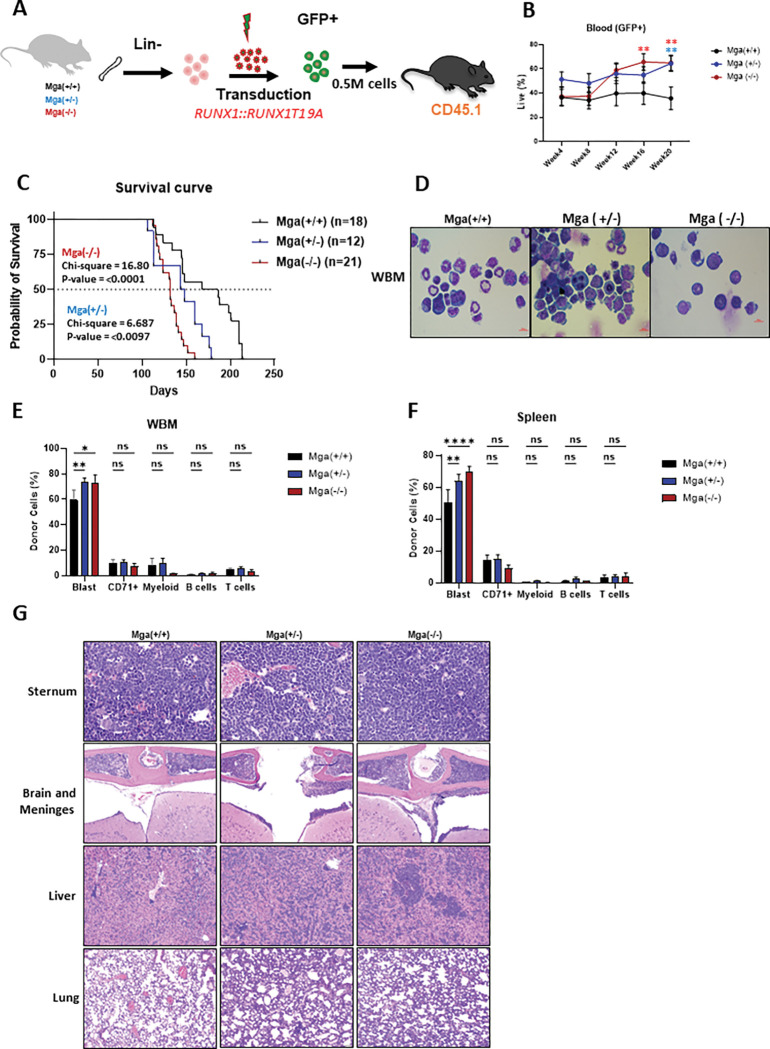
Mga deficiency enhances *RUNX1::RUNX1* Tleukemia development. **A**. Schematic of retroviral transduction of *RUNX1::RUNX1T1 9A* in lineage-negative HSPCs from WT, Mga(+/−), and Mga(−/−) mice (CD45.2) and transplantation into lethally irradiated recipient mice (C57BL/6, CD45.1). **B**. GFP expression in the peripheral blood over time. **C**. Survival curve of recipient mice. Statistics were done using Mantel- Cox test in GraphPad Prism. **D**. Cytospins of whole bone marrow (WBM) harvested at sacrifice stained with Wright–Giemsa; magnification 60X. **E-F**. Flow cytometric analysis of WBM (F) and Spleen (G) harvested from terminal recipient mice stained with tumor panel from Supplemental Table 1. Blast = GFP+, cKit+, lineage-negative. **G**. Hematoxylin and eosin (H&E)-stained sections of sternum marrow, brain meninges, liver, and lungs harvested from indicated terminal recipient mice. Magnification 40X. Statistics: (**B**) One-way ANOVA with Bonferroni correction (* p<0.05, ** p<0.01). Error bars indicate the standard error of the mean from three or more biological replicates. (**F&G**) Two-way ANOVA with Dunnett’s multiple comparisons test.

**Figure 6:**
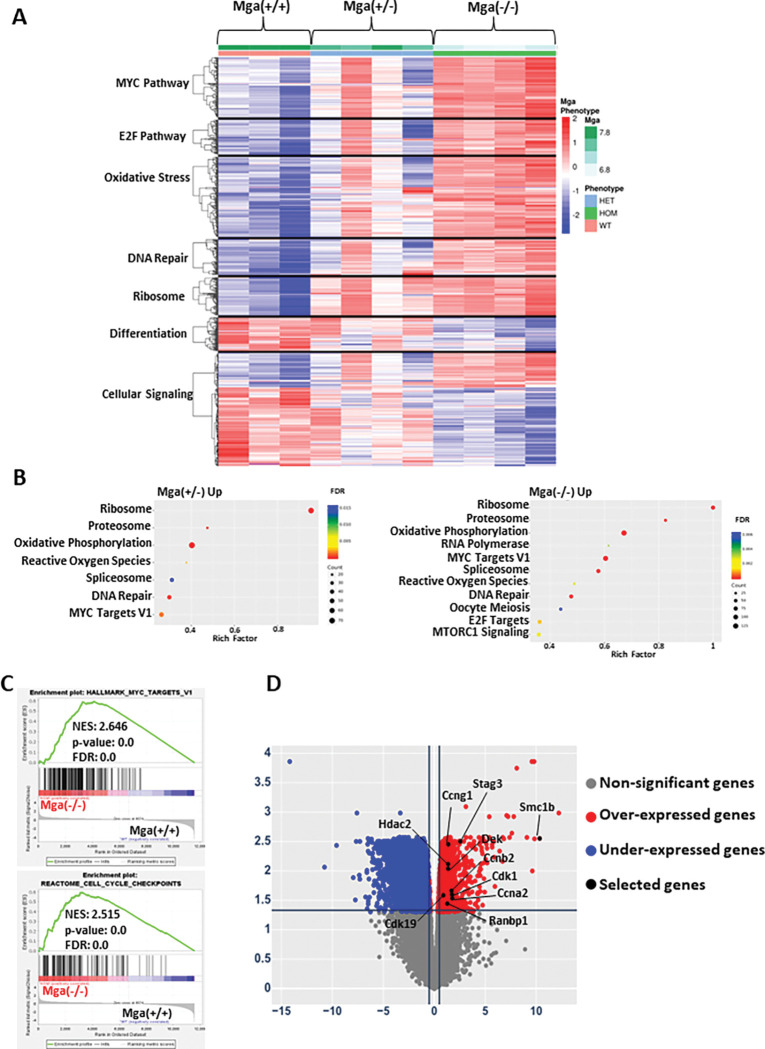
Mga deficient tumors lead to hyperproliferative transcriptional profile. **A**. Heatmap showing the enrichment of differentially regulated pathways in *RUNX1::RUNX1T1 9A* tumors (GFP+) from *WT,* Mga(+/−), and Mga(−/−) cells. The z-scale is set to between 2 and −2; red=upregulation, blue = downregulation. **B**. Rich factor plot of top upregulated pathways from *RUNX1::RUNX1T1 9A* expression in *Mga(+/−)* and Mga(+/−) cells. The size of each dot represents gene count and the color represents FDR. **C**. GSEA enrichment plots of selected upregulated pathways in *RUNX1::RUNX1T1* expressing Mga(−/−) GFP+ cells vs. Mga(+/+) GFP+ cells isolated from spleens. **D**. Volcano plot of differentially expressed genes (DEGs) from *RUNX1 ::RUNX1 T19A* expression in Mga(−/−) vs. Mga(+/+)cells. Statistics: One-way ANOVA with Bonferroni correction (* p<0.05, ** p<0.01). Error bars indicate the standard error of the mean from three or more biological replicates.

These data show that *Mga* deficiency is not sufficient for an overt hematopoietic malignant phenotype, but do suggest that the loss of *Mga* may promote a transcriptional and epigenetic landscape that could enhance the aggressive phenotypes of leukemia in the presence of an oncogenic driver. This was tested using an established murine leukemia model using retroviral expression of *RUNX1::RUNX1T1 9a,* which encodes a shortened form of *RUNX1::RUNX1T1,* that induces the rapid development of AML (Fig. 5A)^22^. *Ex vivo RUNX1::RUNX1T1*9a expression in Mga(+/+), Mga(+/−), and Mga(−/−) HSPCs showed that Mga(−/−) HSPCs have an increase in cell growth when compared to controls, while the Mga(+/−) HSPCs had little to no change, however, both had a significant increase in CFU colony formation and serial replating (**Supp**. Figure 5A-C). These data suggest that *Mga* deficiency may enhance the development of leukemia in a *RUNX1::RUNX1T1 9A* model. This was confirmed *in vivo* as Mga(+/−) and Mga(−/−) HSPCs expressing *RUNX1::RUNX1T1 9A* rapidly expanded and developed into leukemias characterized by immature GFP+, cKit + myeloid progenitor-like cells when transplanted into lethally irradiated recipient mice (Fig. 5C **and Supp**. Figure 5D-E**&H**). *Mga* deficiency in *RUNX1::RUNX1T1 9A* expressing cells led to a significantly shortened leukemia latency with a median survival of 177 days for controls, 146 days for Mga(+/−) mice, and 132 days for Mga(−/−) mice (Fig. 5C). Immunophenotyping of the BM and spleen confirmed that *RUNX1::RUNX1T1 9A* expressing tumors were cKit + and morphologically resembled immature blast cells (Fig. 5D–F **and Supp**. Figure 5F**&G**). Pathological analysis of the bones (sternum), brain and meninges, liver, and lungs further show that *RUNX1::RUNX1T1 9A* expression in Mga(+/−) and Mga(−/−) HSPCs leads to mice that are more severely infiltrated by leukemia compared to control mice (Fig. 5G). Transcriptional analysis by RNA-seq of sorted GFP + *RUNX1::RUNX1T1 9A* expressing tumors from Mga(+/−), and Mga(−/−) moribund mice revealed upregulation of pro-proliferation pathways including MYC, ribosome, and oxidative phosphorylation, while Mga(−/−) tumors interestingly had downregulation of differentiation pathways including angiogenesis and hematopoietic cell lineage (Fig. 6A–C). Mga(−/−) leukemic cells demonstrated a higher expression of *Smc1b* as well as an increased expression of cell cycle-related genes such as *Ccng1, Ccnd3, Cdk1, Cdk19, Dek,* and *Ranbp1* (Fig. 6D)^23,24^. These transcriptional data support our cell growth and serial CFU replating phenotypes. To determine if these tumors were transplantable, *RUNX1::RUNX1T1 9A* expressing tumors were serially transplanted into sublethally irradiated mice. These secondary mice quickly developed AML 4–6 weeks post-transplantation, however, there was no significant difference between Mga(+/+), Mga(+/−), and Mga(−/−) mice (**Supp**. Figure 6A**&B**).

## Discussion

**Figure 7:**
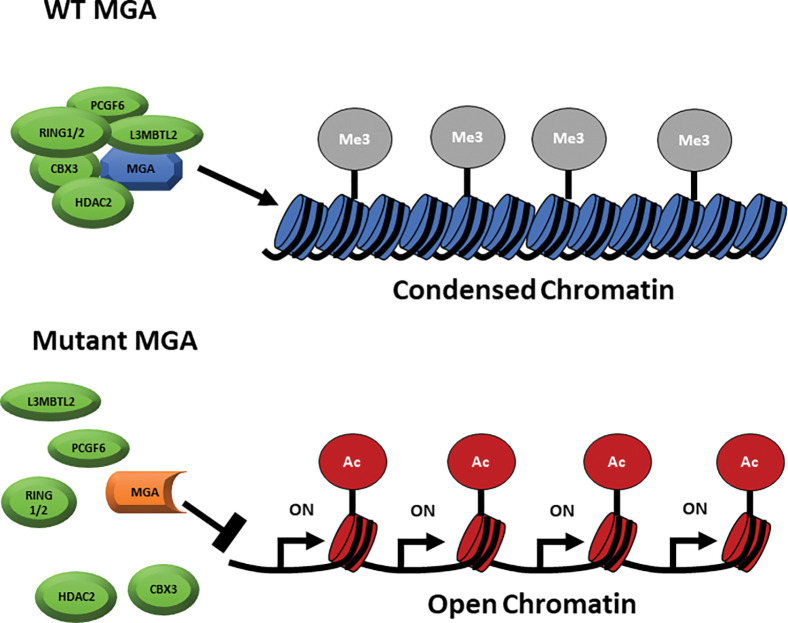
Model of MGA deficiency. **A**. Model of MGA deficiency in hematopoietic cells. The expression of full-length MGA leads to ncPRC1.6 complex formation and the deposition of repressive H3K27me3 of *MYC target* genes leading to a condensed and transcriptionally repressed chromatin status (top). Somatic mutations of *MGAas* seen in AML; lead to the abolishment of ncPRC1.6 complex formation leading to an open and transcriptionally active chromatin status.

Here we generated both human and mouse models to assess the role of MGA in normal and malignant hematopoiesis. Our data support the well-documented antiproliferative role of MGA and show that loss of *MGA* leads to an open and active epigenetic chromatin status, and subsequently the upregulation of several critical pathways for cell growth and proliferation (Fig. 7)^2,3^. Previous reports in non-hematopoietic models show that components of the ncPRC1.6 complex, such as MGA and E2F6, regulate transcriptional control of critical regulators of meiosis such as *STAG3, SMC1B, MEIOC,* and *CNTD1* and overall result in transcriptional silencing ^2,25^. While components of the ncPRC1.6 complex are known to play a critical role in embryonic stem cell maintenance, as depletion of MGA, L3MBTL2, and PCGF6 leads to defects in pluripotency, proliferation, and differentiation, the impact of disrupting this complex has not been thoroughly evaluated in hematopoietic cells^3,6,26^. Our hematopoietic MGA-KO models support these reports, revealing a similar transcriptional landscape, including the increased expression of genes such as *Cdk1, Smc1b,* and *Ccne1,* when *Mga* is disrupted. Ccne1 and Cdk1 are known regulators of cell cycle progression, while Smc1b is critical for genomic stability and is suggested to play a role in cell proliferation^20,21,27,28^. We believe the upregulation of these genes and others is due to the direct loss of MGA-dependent ncPRC1.6 binding and regulation leading to the serial replating and cell growth advantage of Mga(−/−) HSPCs. Overexpression of these genes has also been observed in several cancer types including AML, hepatocellular carcinoma, and ovarian carcinoma^29–31^. The lack of changes in H2AK119Ub1 upon MGA depletion was surprising. However, the decrease in H3K27me3 and increase in H3K27Ac suggest both that there are functional redundancies with other PRC1 complexes and that the binding of MGA-dependent ncPRC1.6 to the loci is more critical for transcriptional repression^32,33^. To further support this, the open chromatin of MGA target genes in MGA deficient cells is likely due to the loss of L3MBTL2, which can regulate chromatin compaction regardless of histone modification status^34^.

Despite these clear transcriptional and epigenetic changes, the loss of MGA in the hematopoietic compartment alone is not sufficient to promote profound hematopoietic defects or the development of a hematopoietic neoplasm, even under stress conditions such as serial BM transplantation and 5-FU treatments (data not shown). These findings support the notion that the loss of function mutations in MGA observed in hematologic malignancies are cooperating mutations that provide a transcriptional state that may enhance the effects of tumor drivers. In support of this hypothesis, the latency of leukemia induced by a viral model of *RUNX1::RUNX1T1 9A* was significantly shortened when expressed in both Mga(+/−) and Mga(−/−) hematopoietic cells, and the resulting leukemic cells displayed a more immature phenotype.

*RUNX1::RUNXT1* AMLs have been shown to be dependent on the gene activation of D cyclins such as *CCND2* and *CCND1*^35*,*36^. Overall, we propose that the shortened latency in our *RUNX1::RUNXT1 9A* model is likely due to MGA-deficient cells being primed for enhanced proliferation via the upregulation of cyclin genes and MYC-targets, as shown by our transcriptional analysis of *RUNX1::RUNXT1 9A* tumors. Consistent with this, our previous work identified gain-of-function mutations in *CCND2in RUNX1::RUNX1T1* AMLs, which has been supported by other studies^37^. The findings presented here suggest that *MGA* loss may phenocopy these *CCND2* mutations. Further supporting this claim is the observation that *MGA* and *CCND2* were mutually exclusive in our previous study^17^. Collectively, our data highlight *MGA* as an important regulator of pathways critical for leukemia development, such as MYC activation, cell cycle, and oxidative phosphorylation, and that loss of function mutations identified in patients can functionally cooperate with the RUNX1::RUNX1T1 oncoprotein^21,38–41^. Importantly, we have also defined the *in vivo* consequences of *Mga* loss by using a conditional knock-out approach that circumvents the embryonic lethality of constitutional knock-out models, thus providing the scientific community with an important model.

## Methods

### Animals

We used Ingenious Targeting Laboratory (Stony Brook, NY, USA) to generate the conditional *Mga* KO model in C57BL/6 mice that bore a LoxP-flanked exon 3 of *Mga* using embryonic stem cell-based gene targeting. CD45.1 and C57BL/6 mice were obtained from Jackson Laboratory. Blood, WBM, and spleens were harvested as previously described^42^. HSPCs were isolated from WBM using EasySep^™^ Mouse Hematopoietic Cell Isolation kit. All Animal studies were approved by St. Jude Children’s Research Hospital Institutional Animal Care and Use Committee.

### RNA-Seq Analysis

MOLM-13 cells or Lin−,cKit + HSPCs were harvested and RNA was extracted using a quick-RNA Microprep kit (Zymo Research, CA). RNA-seq was done using TruSeq Stranded Total RNA library kit (Illumina, CA) as previously described^43^. The RNA-Seq paired-end reads were mapped to the mouse mm10 genome or human hg38 genome using STAR and quantified using RSEM^44,45^. Differentially expressed gene analysis and GSEA was done as previously described^42,46^.

### CUT&RUN Analysis

MOLM-13 cells or Lin−, cKit + HSPCs were harvested at 500,000 cells for each antibody probe. Genomic localization profiling was performed using Cleavage Under Targets and Release Using Nuclease kit (CUT&RUN) according to the manufacture’s protocols (Epicypher, NC) as previously described^47^. Library preparations of up to 6ng of isolated DNA fragments were done using NEBNEXT Ultra library prep Kit with AMPure XP beads (Beckman Coulter, CA) following the NEBNext^®^ Ultra^™^ DNA Library Prep Protocol for Illumina^®^ With Size Selection (E7370) V.2 (fragment size < 70bp). CUT&RUN libraries were sequenced using Novaseq 6000 by performing 100 cycles of paired-end sequencing (200 cycles total). CUT&RUN bam files were pre-processed and peaks were called using methods previously described^48^. Peaks from replicate samples were merged and fragments covering those peaks were counted using bedtools version 2.25^49^. Peaks differentially expressed between treatment groups were determined using a combination of the R tools limma and voom as previously described^46^. Peaks were annotated using HOMER version 4.10^50^. The annotation was simplified by combining the HOMER annotations into 3 categories. Peaks within 3kb of a transcription start site were called ‘TSS +/− 3kb’. Peaks annotated as intergenic were labeled as “Intergenic”. Peaks annotated as non-coding were labeled “non-coding” and all other peaks were labeled ‘Genebody’.

### ATAC-seq Analysis

MOLM-13 cells or Lin−,cKit + HSPCs were harvested at 50,000 cells. Open chromatin status was assessed using the assay for transposase-accessible chromatin via sequencing (ATAC-seq) kit (Active Motif, CA) following the manufacturer’s protocols. Library prep, included in kit, was done following the manufacturer’s protocols. ATAC-seq libraries were sequenced using Novaseq 6000 by performing 100 cycles of paired-end sequencing (200 cycles total). Analysis was done by the Center for Applied Bioinformatics at SJCRH. Raw reads in fastq format were processed with Trim-Galore tool (v0.4.4, Krueger F. (2012)), in order to remove potential adapters and quality trim 3’ end of reads with cutadapt program, followed by FastQC analysis^51,52^. A quality score cutoff of Q20 was used and the first 15bp of each reads were also clipped to reduce the GC bias. Next, reads were mapped to the human reference genome (hg38; GRCh38.p12) with BWA mem (0.7.17-r1188), then converted to BAM format and deduplicated with fq2bam (v3.0.0.6)^53^. Subsequently, uniquely mapped properly paired reads were extracted with samtools (v1.2), and fragments were extracted with bedtools(v2.24.0)^49,54^. Chromatin status (open/closed) was defined by a q-value of ≤ 0.05 and a log2FC of +/−2 compared to indicated WT control.

## Data Availability

RNA-seq, CUT&RUN, and ATAC-seq data will be deposited into Gene Expression Omnibus (GEO) (accession number, pending).
